# Single seeds exhibit transcriptional heterogeneity during secondary dormancy induction

**DOI:** 10.1093/plphys/kiac265

**Published:** 2022-06-07

**Authors:** Michal Krzyszton, Ruslan Yatusevich, Magdalena Wrona, Sebastian P Sacharowski, Dorota Adamska, Szymon Swiezewski

**Affiliations:** Laboratory of Seeds Molecular Biology, Institute of Biochemistry and Biophysics, PAS, Warsaw 02-106, Poland; Laboratory of Seeds Molecular Biology, Institute of Biochemistry and Biophysics, PAS, Warsaw 02-106, Poland; Laboratory of Seeds Molecular Biology, Institute of Biochemistry and Biophysics, PAS, Warsaw 02-106, Poland; Laboratory of Seeds Molecular Biology, Institute of Biochemistry and Biophysics, PAS, Warsaw 02-106, Poland; Genomics Core Facility, Centre of New Technologies, University of Warsaw, Warsaw 02-097, Poland; Laboratory of Seeds Molecular Biology, Institute of Biochemistry and Biophysics, PAS, Warsaw 02-106, Poland

## Abstract

Seeds are highly resilient to the external environment, which allows plants to persist in unpredictable and unfavorable conditions. Some plant species have adopted a bet-hedging strategy to germinate a variable fraction of seeds in any given condition, and this could be explained by population-based threshold models. Here, in the model plant Arabidopsis (*Arabidopsis thaliana*), we induced secondary dormancy (SD) to address the transcriptional heterogeneity among seeds that leads to binary germination/nongermination outcomes. We developed a single-seed RNA-seq strategy that allowed us to observe a reduction in seed transcriptional heterogeneity as seeds enter stress conditions, followed by an increase during recovery. We identified groups of genes whose expression showed a specific pattern through a time course and used these groups to position the individual seeds along the transcriptional gradient of germination competence. In agreement, transcriptomes of dormancy-deficient seeds (mutant of *DELAY OF GERMINATION 1*) showed a shift toward higher values of the germination competence index. Interestingly, a significant fraction of genes with variable expression encoded translation-related factors. In summary, interrogating hundreds of single-seed transcriptomes during SD-inducing treatment revealed variability among the transcriptomes that could result from the distribution of population-based sensitivity thresholds. Our results also showed that single-seed RNA-seq is the method of choice for analyzing seed bet-hedging-related phenomena.

## Teaser

Single-seed RNA sequencing enables analysis of gene expression variability and elucidates secondary dormancy induction as a potential bet-hedging strategy.

## Introduction

In most plant species, mother plants produce large numbers of seeds that undergo dispersion, allowing colonization of new territories and propagation into further seasons. Seeds can postpone germination even under an ideal combination of environmental conditions in a phenomenon called seed dormancy (Nonogaki, 2014). Seeds of many species acquire primary dormancy upon maturation, which is later released to enable seedling establishment. The delay of germination is crucial for the survival of fragile seedlings in environments with contrasting seasons or highly changeable weather. In addition, sessile seedlings can encounter unpredictable risks such as herbivores and drought, from which they are generally more protected as seeds. That is why choosing the right time to germinate may be a matter of life and death. Multiple studies of that phenomenon, including in the model plant Arabidopsis (*Arabidopsis thaliana*), showed substantial variability of dormancy depth even between genetically identical seeds (Mitchell et al., 2016). Differences between seeds underlay bet-hedging strategy that maximizes the chances of survival of the seed pool in the long term. Variability in seed germination could be explained by population-based threshold (PBT) models. In PBT models, sensitivity for external conditions and hormone levels show the normal distribution in the population, and germination of a seed is only possible if these thresholds are achieved. Moreover, when sensitivity thresholds are surpassed, the speed of germination is proportional to the difference between the detected level of the factor and the threshold (Bradford and Trewavas, 1994; Bradford, 2018). Given the fact that Arabidopsis is a predominantly self-pollinating plant and laboratory plant lines are homozygous, seeds produced by one mother plant are genetically identical (Meyerowitz, 1989). Resulting genetic homogeneity suggests that in some cases, germination time variability described with distributions of PBTs could be mediated by the genes’ expression diversity. On the molecular level single-seed variability was studied in terms of endo-β-mannanase enzymatic activity (Still and Dahal, 1997), oxygen consumption (Bello and Bradford, 2016), hormone levels (Kanno et al., 2010), and sensitivity (Bradford and Trewavas, 1994). However, single-seed variability was never assayed with genome-wide transcriptomic analyses. In *A. thaliana*, transcriptomic studies of seed-related processes are limited to seed pools due to the very small size of a single seed and the high library preparation costs. These experiments revealed a multilayer mechanism of seed dormancy and germination time control, including hormonal regulation by ABA (Abscisic acid)  and gibberellin (Carrera-Castaño et al., 2020) and key regulatory genes like *DELAY OF GERMINATION 1* (*DOG1*; Bentsink et al., 2006). However, because of the technical limitations, these studies are not informative about the seed variability, its extent, sources, and mechanisms.

Secondary dormancy (SD) induction is a natural process in which germination competent seeds that lost primary dormancy enter a reversible dormant state due to environmental stress conditions (Buijs, 2020). In terms of PBT models, SD induction in the seed population can be interpreted as changes in sensitivity thresholds’ distributions (Bradford, 2005, 2018). This could include both increases in the distribution means and an effect on the variance of the sensitivity threshold distribution. As a consequence, SD induction leads to lower and slower germination even in conditions that ensured uniform germination before stress. While less studied, analysis of SD has some technical advantages over assays of primary dormancy (which are much more difficult to control). Moreover, analysis of SD provides the possibility to conduct several physiological and molecular tests on large and uniform seed pools (Ibarra et al., 2016). From the physiological point of view, SD is fundamental for dormancy cycling and functions of the so-called soil seed banks (Footitt et al., 2011).

Here, we developed a protocol for effective and cost-efficient single-seed transcriptomic analysis and showed its potential to reveal differences between genetically identical seeds that follow the same treatment. As a proof of concept, we assayed a few hundred seeds during SD induction and subsequent seed germination in *A. thaliana*. We observed that stress treatment results in a drop in transcriptome variability between seeds that is rapidly restored and enhanced after transfer to permissive conditions. As a consequence, the level of transcriptional commitment to germination or the dormancy state is better visible after and not during stress treatment. Analysis shows that seeds’ heterogeneity can be at least partially explained by a gradient of transcriptional competence to germinate. This observation was supported by *dog1* mutant single-seed analysis that showed a shift toward the higher values of transcriptional competence to germinate.

## Results

### SD induction

Several protocols exist to induce SD in Arabidopsis (Buijs, 2020). Their application usually leads to a dormancy state in a fraction of stress-treated seeds as revealed by their subsequent incubation in permissive conditions. We have implemented an SD induction method on fully matured Arabidopsis seeds that have lost primary dormancy (due to after-ripening) using a dark and high-temperature treatment as described before (Ibarra et al., 2016).

**Figure 1:**
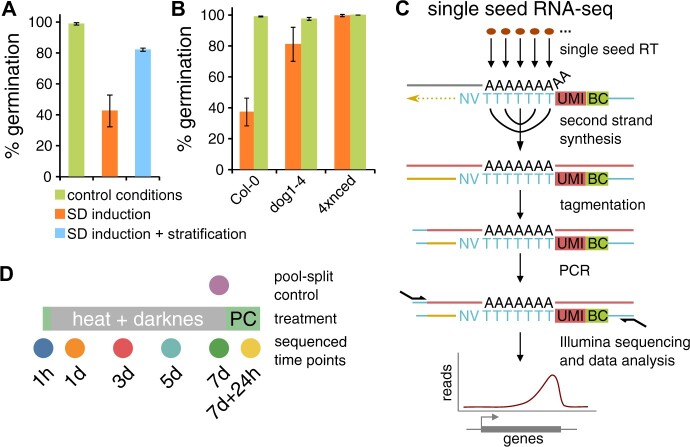
SD induction and single-seed RNA-seq method. A, Comparison of Col-0 seed germination in permissive conditions without pretreatment, after 7 days of SD induction, and after 7 days of SD followed by stratification. B, Seed germination of Col-0, *dog1-4*, and *nced/2/5/6/9* (labeled 4xnced on the plot) after 7 days of SD induction. A and B, Germination of at least 150 seeds was scored after 7 days of incubation. Error bars show standard deviation of three biological replicas. C, Schematic of single-seed RNA-seq protocol. Multiple seeds (brown ovals) are crushed, and polyadenylated mRNA (gray line) is reverse transcribed (RT; yellow arrow line) using oligo(dT) with UMI, BC, and Illumina primer binding site (blue line). After this step, RT reactions are pooled (single arrows from this point). During second-strand synthesis RNA is replaced with DNA strand (red line) and then tagmentation is performed introducing Illumina primer binding sites (blue lines). Final PCR (primers showed as semi-arrows) leads to library amplification enriched at 3′-ends of the genes as shown at the bottom. D, Single-seed RNA-seq SD induction experiment layout. PC and green color rectangles denote permissive conditions. Pool-split control (purple dot) was performed for 7-days time point.

These conditions resulted in a stable pause in germination despite favorable conditions (constant light, 22°C) that was first detected in Col-0 seeds treated for 1 day (4%–20% seeds dormant) and reached 51%–78% of seeds after 7 days of treatment (Figure 1A; and Supplemental Figure S1). The seed germination was entirely restored after standard dormancy release treatment (i.e. stratification—cold treatment), which showed that nongerminating seeds are viable (Figure 1A). Importantly, our protocol results in a stress-induced inhibition of germination that is dependent both on ABA hormone and DOG1 protein, as shown by assaying SD induction in ABA biosynthesis-deficient quadruple *nine-cis-epoxycarotenoid dioxygenase 2/5/6/9* mutant (*nced2/5/6/9*; Chauffour et al., 2019) and *dog1-4* (primary dormancy defective; Bentsink et al., 2006) mutant seed pools (Figure 1B). The lack of SD induction in these mutants confirmed that our treatment results in a genuine dormancy phenotype and supports the previously described role of DOG1 in that process (Footitt et al., 2011, 2020; Buijs et al., 2020). To better understand dormancy state induction by our protocol, we assayed the germination potential at varying temperatures during recovery after different times of SD induction (0, 1, 3, and 7 days; Supplemental Figure S1). The most obvious observation is an increase of nongerminated seeds fraction at 22°C and 27°C along with longer times of stress treatment. While 32°C is too high for germination for most of the seeds even in control conditions, almost 100% germination at 12°C and 17°C for all tested time points again confirm that seeds are viable after treatment. We also observed a delay in seed germination at 22°C along with the increased time of stress treatment. These results could be described in terms of PBT models as a change in the distribution mean and/or variance of temperature sensitivity thresholds in tested seed pools caused by incubation under stress conditions. However, how these changes are reflected on the transcriptomic level is not clear.

The relationship between seeds’ transcriptomes and PBT models-based control of germination may be complex because of possible reciprocal influence on each other. While we hypothesized that gene expression patterns among single seeds are underlying the distribution of sensitivity thresholds, they may be also intertwined with gene expression changes caused by the response to the environment. This could be even more complicated if some additional external factors like persisting stress are involved.

Apart from the causal link, we expected that seeds transcriptome changes upon SD-inducing stress treatment will reflect a shift of sensitivity thresholds and resulting germination competence. This would be observed as an effect on the expression of genes involved in the positive and/or negative regulation of seed germination.

As only a fraction of seeds abstains from germination in a specific temperature range, we also expected that extent of transcriptomic variability between seeds may change upon treatment. Gene expression diversity may be influenced by multiple external and internal factors. On the one hand, we observe the appearance of two phenotypically different seed types (germinating and dormant) so the increase in seed transcriptomic variability is the most probable outcome. This could reflect changes in the variance of sensitivity thresholds and/or resulting germination competence distributions. However, prolonged stress was reported to cause a very strong transcriptomic response (Rasmussen et al., 2013), which possibly could make seeds’ mRNA pool more uniform when compared to initial conditions.

### Single-seed RNA-seq

To study transcriptional heterogeneity changes in a pool of genetically identical seeds, we created a single-seed RNA-seq protocol for Illumina sequencing of mRNA 3′-end fragments from single seeds. Briefly, this approach skips the RNA isolation step and uses barcoded oligo(dT) primers with unique molecular identifiers UMIs to prime cDNA synthesis from raw seed extracts. Overhang present on the reverse transcription primer is used for final amplification which specifies library production to 3′-ends of poly(A)+ RNA species, allowing us to avoid DNA and rRNA depletion steps. As a consequence, analysis is more quantitative than qualitative with one read detected per mRNA molecule after the PCR deduplication step was performed, thanks to UMI sequences. Although crucial for parallel processing of dozens of seeds, reverse transcription performed directly on seed extracts excludes from detection mRNAs expressed at low levels due to suboptimal reaction conditions. Dozens of reverse transcription reactions may be pooled due to the presence of unique barcodes which substantially reduces reagent consumption and work time. Further steps consist of cDNA second strand synthesis by nick translation, tagmentation with homemade Tn5 enzyme, and finally PCR amplification of libraries with standard Illumina primers (Figure 1C). Our protocol enables fast and cost-effective transcriptome assaying for hundreds of Arabidopsis seeds in a single experiment.

We performed single-seed RNA-seq on Col-0 seeds during SD induction at six time points (Figure 1D) on one of the seed pools tested for dormancy states (marked on Supplemental Figure S1). Seeds imbibed for 1 h were used as a proxy for untreated seeds, followed by 1, 3, 5, and 7 days of treatment. Finally, 7-day incubated seeds followed by 24 h in permissive conditions were used to assess the early steps of seed recovery and germination. Importantly, no seeds at this time point of recovery show visible signs of germination (Supplemental Figure S1D). To estimate technical variability, we used 7-day-treated seeds and performed a pool-split experiment in which single-seed extracts were mixed, split into separate reactions, and processed as all other samples. Each time point consisted of 96 seeds and was divided into three technical replicate seed batches. We excluded low-quality seeds (<5,000 UMIs) and lowly expressed genes (<1 read per seed on average), which resulted in 659 seeds and 8,687 genes after initial filtering. We observed substantial differences between libraries from different time points regarding the numbers of sequenced reads and identified genes (Supplemental Figure S2A). One source of these differences may be the lower quality of some libraries, which we quantified by counting reads mapped outside nuclear protein-coding genes. Our single-seed RNA-seq method is designed to detect only poly(A)+ RNA species. That is why we expect that only reads mapping to genes producing such RNA molecules (almost exclusively nuclear-encoded mRNAs) are specific. In such a scenario, reads mapping to intergenic nuclear regions, nonprotein-coding genes, as well as organellar genomes, should be considered unspecific and their accumulation may reflect inefficient reverse transcription. These unspecific reads are mostly primed by oligo(dT) primers as they preserve the expected structure of the R1 read (Supplemental Figure S2B). Importantly, we observed that the number of genic and unspecific reads are negatively related (Supplemental Figure S2C), and unspecific reads accumulate in defined positions (Supplemental Figure S2, D and E). We believe that inefficient reverse transcription leads to the production of DNA templated products from regions of higher DNA accessibility present in crude seed extracts. Localized accumulation of unspecific reads allowed us to filter out genes whose read count may be affected by this unspecific background. We removed genes with the read number correlated (Pearson correlation >0.3) with the amount of intergenic signal (Supplemental Figure S2, F and G). As expected, the distribution of the reads of 6,430 remaining genes showed a clear bias toward the 3′-end of genes in all time points (Supplemental Figure S2H). In summary, this showed that our method can interrogate hundreds of single-seed transcriptomes in 2–3 days of work required for library preparation.

**Figure 2:**
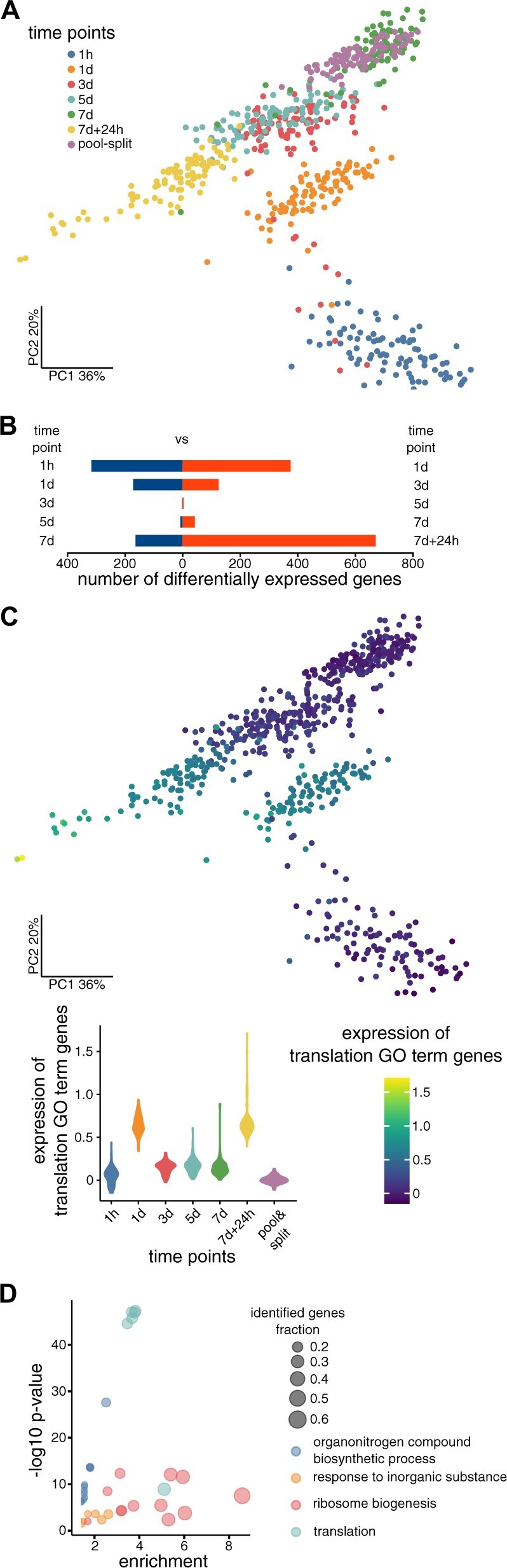
Single-seed RNA-seq separates seeds according to SD induction time. A, PCA map of single seeds (*n* = 659) created using Seurat package with sctransform normalization. B, Number of DEGs between sequential time points (Seurat FindMarkers Wilcoxon Rank Sum test; adjusted *P*-value < 0.05; |log2FC| > 1). Blue and red colors denote the decrease and increase of gene expression, respectively. C, PCA map with Expression signature (mean normalized expression) of genes belonging to GO term “translation” (GO:0006412) created using Seurat AddModuleScore. The spread of values is shown as a violin plot (data were sctransform normalized). D, GO terms enrichment analysis results for the 500 genes most affected in the time course (gprofiler2 R package, background limited to 6,430 genes analyzed in the experiment—Supplemental Data Set 1, reduction of GO terms using rrvgo R package). Colors of the bubbles show parent GO terms (biological process) and their size reflects the fraction of genes for a given term among the 500 genes most affected in the time course.

### Gene expression changes during SD induction

The obtained read count matrix was used to perform a detailed analysis of seed transcriptomes with the Seurat library and sctransform normalization (Hafemeister and Satija, 2019; Hao et al., 2021). We selected this method as it not only allows for normalization and variance stabilization of sparse molecular count data but also enables adding additional covariates to the model, which in our case includes the number of unspecific reads per seed and the presence of technical replicate seed batches. In this way, we are able to normalize our data along with the removal of confounding technical variability while preserving true biological variation. A principal component analysis (PCA) map showed seeds grouped according to experimental time points (except 3 and 5 days, which group together; Figure 2A), suggesting that biological treatment was the primary driving force behind the observed differences among seed transcriptomes. We confirmed that technical parameters like total read count, intergenic reads, detected genes number, and seed batches did not contribute to the relative position between groups of seeds. Nevertheless, seed batches and the number of intergenic reads did, to some extent, confound seed position inside groups (Supplemental Figure S3, A–D). Differential gene expression analysis identified a few to several hundred genes that changed their expression between sequential SD induction time points (Figure 2B). Short 1-day SD induction decreased the expression of 316 mRNAs, including abiotic stimulus response-related genes (hypoxia, water, osmotic, and temperature) as revealed by GO-term (gene ontology) analysis (Supplemental Figure S3E). Notably, many genes with expression upregulated by 1-day treatment are mainly involved in translation and ribosome biogenesis (Supplemental Figure S3F). The same group of genes was downregulated during the transition from 1 to 3 days and finally upregulated upon seeds recovery in 7 days + 24-h time point (Supplemental Figure S3F). This observation agrees with recent work showing that the expression of translation and ribosome-related genes can serve as a marker for germination competence (Buijs et al., 2020). In our experiment, a 1 day SD induction led to activation of these genes, presumably because it involved the imbibition of nondormant seeds. However, prolonged stress conditions turned off their expression, which was finally re-established during seed recovery (Figure 2C). In accordance with the role of translation-related genes in seed biology during SD induction, these genes are enriched among the top 500 most time course affected genes (Figure 2D). In line with the published work (Wilhelmsson et al., 2019; Buijs et al., 2020), our data show that translation is an important player in germination regulation in seeds.

**Figure 3:**
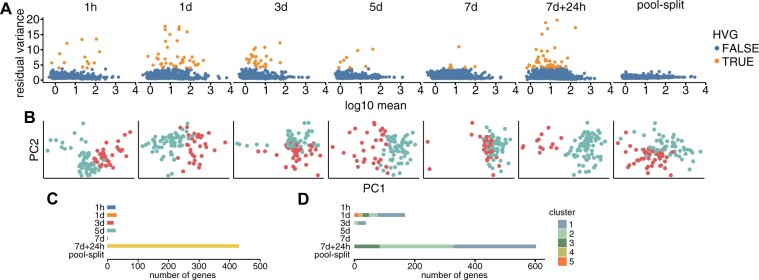
Seeds show the highest transcriptome variability at the recovery time point and lowest at the final time point of stress treatment. A, Plots of gene residual variances and gene expression levels after the sctransform normalization with HVGs highlighted. HVGs are defined as those with residual variance >4 (one unit above the maximum residual variance of any gene from pool-split control). B, PCA maps of seeds sub-pools identified using Seurat FindClusters with resolution set to obtain two groups (red versus teal color). C, Number of DEGs between seed sub-pools (Seurat FindMarkers Wilcoxon Rank Sum test; adjusted *P* < 0.05; |log2FC| > log2(1.5)). D, Number of genes in co-expressed genes groups at each time point. Gene expression correlation among seeds was calculated (scran R package), and gene pairs with correlation >0.5 were used for clustering (RBGL R package). Shown are gene clusters with at least 10 genes.

Our analysis of SD induction (Supplemental Figure S1) suggested a gradual increase in dormancy levels during treatment. Such an effect will likely be linked with a specific group of genes whose expression would be correlated (negative regulators of seed germination) or anticorrelated (positive regulators of seed germination) with observed seed dormancy levels. To identify such genes we first performed differential gene expression analysis between 1, 3, 5, and 7-day-treated seeds. Scaled expression of a subset of affected genes was clustered to identify groups of genes behaving in a concordant manner (Supplemental Figure S3G). We were interested in genes showing gradual upregulation (Group 1) or gradual downregulation of expression (Group 9). As seeds in the 1-day time point still show imbibition-related activation of some genes (Supplemental Figure S3F) those showing a concordant change in expression with exception of this time point may also directly mirror dormancy levels (Groups 7, 8, and 10). Among identified genes (Supplemental Data Set 1) we noticed the presence of several well-known seed germination regulators including *INCREASE IN BONSAI METHYLATION 1* and *ABSCISIC ACID RESPONSIVE ELEMENT-BINDING FACTOR 1* (*ABF1;* Group 1), *GIBBERELLIN 20-OXIDASE 3* (*GA20OX3;* Group 9) and *SOMNUS* (*SOM*) (Group 7; Kim et al., 2008; Nonogaki, 2014).

### Prolonged stress conditions result in reduced seed transcriptome variability

To identify potential variability between seeds inside the time points, we analyzed our data for each treatment separately with reselection of expressed genes (at least 1 UMI on average per seed) and removal of batch effects. Plots of residual variance show that the sctransform procedure successfully removed dependency between variance and gene expression and demonstrate a minimum for pool-split control (Figure 3A). We defined highly variable genes (HVGs) as those with residual variance >4 (1 unit above the maximum residual variance of any gene from pool-split control). Interestingly, the number and residual variance of such defined HVGs was lowest for both 5 and 7 days among all time points (Supplemental Figure S4A). From the total of 99 HVGs identified in all time points, 26 genes are shared between at least two conditions (Supplemental Figure S4B). Among HVGs there is a substantial number of genes encoding seed storage materials or late embryogenesis abundant proteins, in addition, the 7 days + 24-h time point is also characterized by expression variability of genes involved in translation (Supplemental Data Set 1).

**Figure 4:**
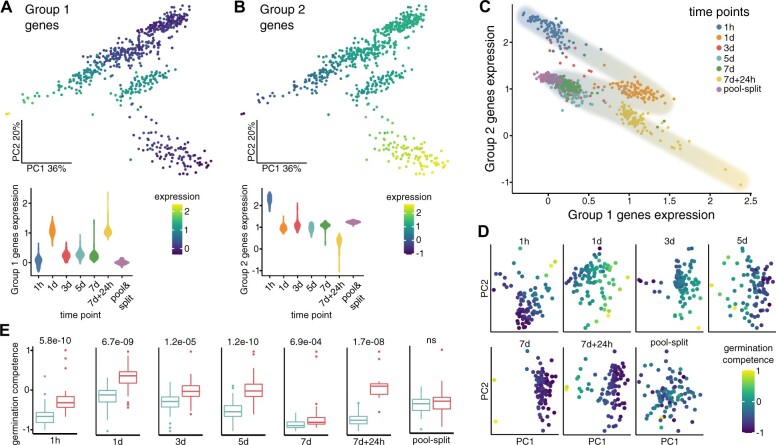
Co-expressed gene groups align seeds on the path toward germination. A and B, Gene expression signature values (mean normalized expression) of two main globally co-expressed gene groups overlaid on the PCA map of the time-course experiment. Globally co-expressed gene groups were identified as described in Figure 3D, except that all seeds (without pool-split control) were used. Identified groups were used to calculate signatures (Seurat AddModuleScore). Signature values spread shown as a violin plot (data were sctransform normalized). C, Plot of Groups 1 (germination-associated) and 2 (dry seed-associated) gene expression signatures values for each seed. The proposed transcriptomic germination competence path is illustrated as shading from blue (more dormant seeds) to yellow color (germination competent seeds). D, Germination competence signature overlaid on PCA maps for each time point. Composite signature values were calculated using Vision R package based on the expression of Groups 1 and 2 genes and scaled to the −1 to 1 range. E, Boxplots for each time point showing values of composite signature for seeds sub-pools (red and teal color match two clusters from Figure 3B). *P*-values of Wilcoxon rank sum test for comparison of sub-pools are shown above plots. Boxplots center lines show the position of the median, box limits show upper and lower quartiles, whiskers show a 1.5 interquartile range, and outliers are marked with dots. The color code for each treatment is provided in the figure.

We expect that meaningful expression variability could lead to the formation of seed sub-clusters with coherent gene expression patterns. Therefore, we performed seed clustering into sub-pools for each time point, followed by differential gene expression analysis between them (Figure 3B). The division into two sub-pools was chosen to keep seed groups numerous enough to perform robust differential gene expression analysis. As the number of germinating seeds is affected by both the time of SD induction and recovery temperature (Supplemental Figure S1), we cannot expect that number of seeds in sub-pools identified during the treatment will correspond directly to the final fraction of germinating seeds. The highest number of differentially expressed genes (DEGs) was identified for two clearly separated seed groups in the 7 days + 24-h time point, and those genes are again mostly related to translation (Figure 3C; Supplemental Data Set 1). Other sub-pools of seeds can be distinguished by far fewer genes, with zero genes for pool-split control. Importantly, levels of variance explained by two main principal components also show specific patterns among analyzed time points, suggesting a higher uniformity of gene expression in 5- and 7-day time points where variance is more evenly distributed among PCs (Supplemental Figure S4C).

Another way to estimate gene expression variability and its coherence between seeds is to identify groups of genes with highly correlated expression. We calculated pairwise gene expression correlations (scran R) and selected gene pairs with a correlation >0.5. On this set of gene pairs, we performed graph clustering using the presence of correlation as the edge between two genes (RBGL R). Finally, we filtered obtained gene groups to contain at least 10 genes, which resulted in the identification of five gene groups for 1-day time point, three groups for 7 days + 24 h, two groups for 3 days, and no group for 1 h, 5 days, 7 days, and pool-split control (Figure 3D). Again, translation-related genes account for the largest groups with a correlated expression both for 1 day and 7 days + 24-h time points (Supplemental Data Set 1). Interestingly, *DOG1*, *REDUCED DORMANCY 5* (*RDO5*), and *SOM*, three genes important for seed dormancy regulation (Bentsink et al., 2006; Kim et al., 2008; Xiang et al., 2014), were found in gene Group 3 for 7 days + 24-h time point (Supplemental Data Set 1), showing that observed gene co-expression networks are not restricted to translation factors. Out of 99 HVGs identified in total in different time points, 84 were also included in these co-expressed gene groups (Supplemental Figure S4D).

Based on the results of germination tests, we expected that single-seed transcriptomic analysis would show some initial variability of the seed pool that would be changed upon stress treatment. It could be increased if germination-related transcriptomic effects prevailed upon stress treatment or decreased if stress-related response uniformized seed population. We also supposed that in both scenarios, extremes of seed transcriptome distribution would point to less-dormant and more-dormant seeds, which is phenotypically demonstrated in permissive conditions. Our observations—the number of DEGs between seed sub-pools, and clustering of gene expression into groups—show higher variability in earlier time points and a decrease in variability during the longest stress condition time point used. Such transcriptomic homogenization upon stress treatment could be explained by the prioritization and strong induction of pathways important for seed survival. Heterogeneity, however, becomes rapidly restored and enhanced after transfer to permissive conditions. The highest variability of seeds from the 7 days + 24-h time point is explained by the presence of two groups of seeds: germinating and dormant which only emerge after stress alleviation. This late differentiation is in agreement with the fact that the number of seeds abstaining from germination after the SD induction protocol can be modulated by the temperature encountered during the recovery phase. Importantly, for all tested time points, variability was higher than in pool-split control, which validates our method as capable of detecting biological differences between individual seeds. To sum up, co-expression and differential expression of genes in seed sub-pools revealed a reduction of seed heterogeneity during persisting stress conditions, followed by seed separation into two subgroups during stress recovery.

### Sources of transcriptional heterogeneity

Several genes important for hormonal regulation of seed biology were found in our experimental data set (Supplemental Figures S3G and S5, A–K). *ABA-RESPONSE PROTEIN* (*ABR*) and *PYR1-LIKE 4* genes as well as several genes encoding seed storage materials or late embryogenesis abundant proteins were among highly confident HVGs. Lowering the threshold of residual variance to 2 in this analysis provided a few more known germination regulators: *RGA TARGET 1*, *ABA REPRESSOR1*, *GIBBERELLIC ACID INSENSITIVE*, *REPRESSOR OF GA 1(RGA1)*, and *XERICO* (Supplemental Data Set 1).

Other factors show lower variability of expression in specific conditions; however, they were identified as differentially expressed between subsequent experimental time points. These include ABA related factors like *ABA-HYPERSENSITIVE GERMINATION 1* (DEG between 1 h and 1 day; Née et al., 2017), *ABA INSENSITIVE 3* (*ABI3*) and *ABI5* (DEG between 1 h and 1 day; Nonogaki, 2014), genes involved in gibberellin-mediated regulation like *GA20OX3* (multiple comparisons; Nonogaki, 2014), *RGA-LIKE 2* (DEG between 1 h and 1 day; Lee et al., 2010), and *ETHYLENE-INSENSITIVE 3* gene (involved in ethylene response; Corbineau et al., 2014). In our experiment, we also identified a set of genes implicated in various aspects of seed biology, including *HEAT SHOCK TRANSCRIPTION FACTOR A9*, *ATP-BINDING CASSETTE F1*, *RDO5*, *ABF1, SOM*, *MOTHER OF FT AND TFL1*, *AGAMOUS-LIKE 67*, *OLEOSIN 1*, and *CRUCIFERINA 1* (Chibani et al., 2006; Siloto et al., 2006; Kim et al., 2008; Xi et al., 2010; Li et al., 2020; Supplemental Figure S5, A–K; Supplemental Data Set 1), as those whose expression is clustered in specific groups. The patterns of these genes’ expression in our time course experiment suggest their role in the regulation of SD.

**Figure 5:**
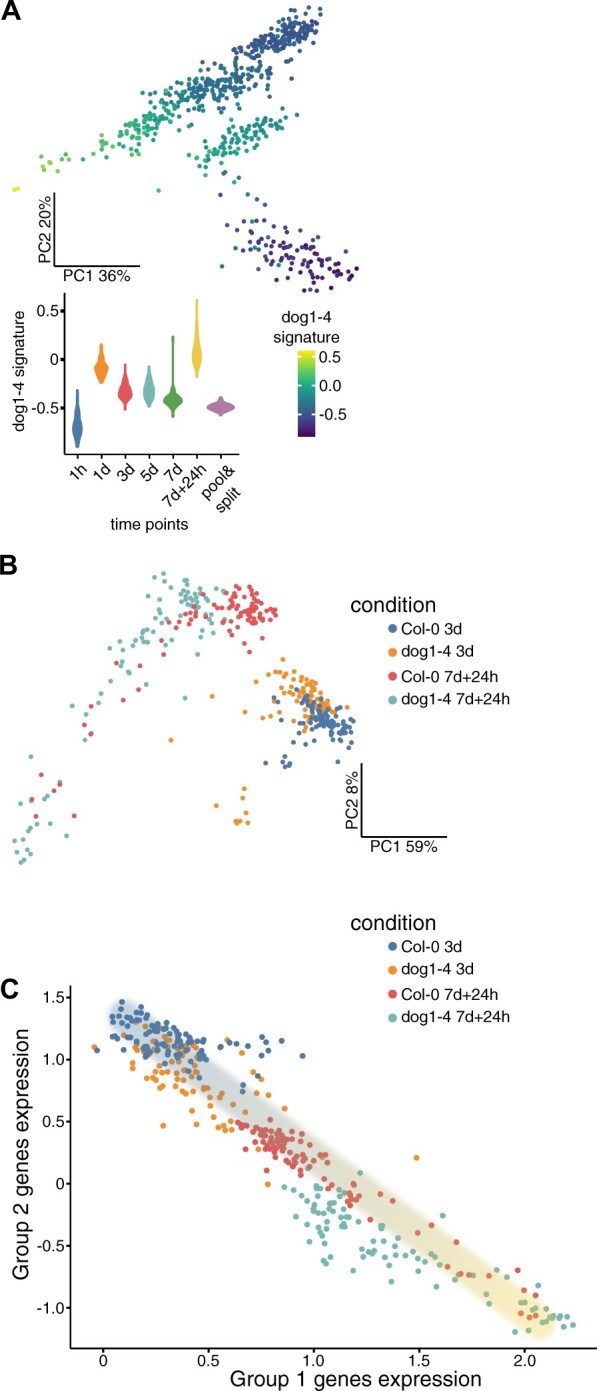
*dog1-4* mutant seeds show higher germination competence. A, *dog1-4* signature overlaid on PCA map of the time-course experiment. Signature values were calculated using Vision R package based on genes differentially expressed between Col-0 and *dog1-4* mutant seeds (mean normalized expression of genes with upregulated and downregulated genes having positive and negative input, respectively). Spread of signature values shown as a violin plot (data were sctransform normalized). B, PCA map of single seeds (*n* = 382) from Col-0 and *dog1-4* in two time points, created using Seurat package with sctransform normalization. C, Plot of Group 1 (germination-associated) and Group 2 (dry seed-associated) signatures values for each Col-0 and *dog1* seed based on globally co-expressed gene groups identified in this experiment, and values of signatures calculated as described in Figure 4. The proposed transcriptomic germination competence path is illustrated as shading from blue (more dormant seeds) to yellow color (germination competent seeds).

To better understand gene expression changes during seed dormancy induction and seed germination, we performed global gene clustering based on their co-expression in each seed from all time points (except pool-split) as described above. We identified five highly interconnected gene groups consisting of 266, 123, 74, 36, and 13 genes, respectively (Figure 4, A and B; Supplemental Figure S6, A–C). Using gene expression from the globally co-regulated gene groups, we calculated their expression signature values (average normalized expression of genes from the group) and overlaid them on the PCA map. Each gene group showed a specific expression pattern during SD induction and a different degree of variability in each of the analyzed time points (Figure 4, A and B; Supplemental Figure S4, A–C; Supplemental Table S1). Groups 1 and 2 genes had largely opposing patterns of expression among seeds. Group 1 genes had the highest expression in 1 day and 7 days + 24-h time points, with few seeds from that last time point having clear separation from the rest. This group consists of genes with enrichment of translation and ribosome-related GO terms (Supplemental Figure S6D). Group 2 genes had the highest expression in 1 h and lowest in 7 days + 24-h time point (Figure 4B). Genes constituting this group included seed storage albumins, oleosins, and some HSP17 family members, all of which accumulate at high levels in dry seeds (Nakabayashi et al., 2005). Groups 3 and 4 included genes whose expression increased during SD induction with a further drop or increase during recovery, respectively. Group 3 included a few transcription factors like ABF4 (Dekkers et al., 2016) and *EBP* (Narsai et al., 2017), and Group 4 included *FIB2* and *NUC-L1* genes, which are crucial for nucleolus functions (Morris, 2008).

**Figure 6:**
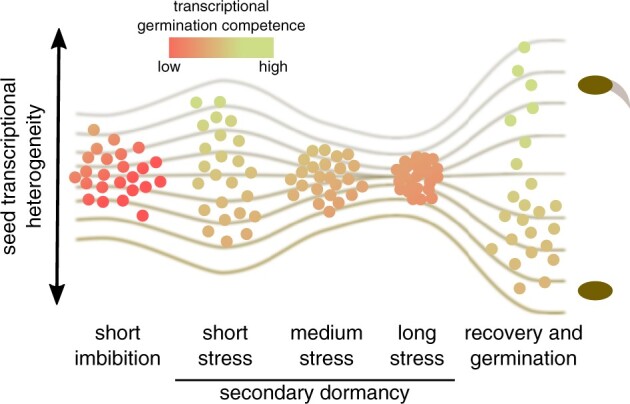
Transcriptional heterogeneity of single seeds during the SD induction. Seed transcriptional heterogeneity is shown as a spread on the vertical axis. The horizontal axis represents the selected time points of our experiment. Seeds are colored according to germination competence, showing higher values in time points physiologically closer to seedling establishment. Lines represent the model of a preset germination decision (described in the text). They link seeds with differential behavior during the recovery phase with untreated (short imbibition) seeds with initially varying germination competence. This model assumes that the relative position of the seeds is maintained throughout the time course.

The above analysis of globally co-regulated gene groups allowed us to visualize a positional relationship between single seeds in our experiment. Patterns of co-expressed genes that belong to the most numerous Group 1 (germination-associated) and Group 2 (dry seed-associated; Figure 4C) align the seeds along a SD induction and recovery path. We hypothesized that the right-most seeds with high expression of translation-related genes represented those most committed to germination. Inspection of the plot indicated heterogeneity, which positions seeds belonging to the same time point along the gene expression gradient as most evident for the recovery phase (Figure 4C). To further substantiate this observation, we used the Vision R package (DeTomaso et al., 2019) to compute the composite gene expression signature of largely antagonistic Groups 1 and 2 genes. In that case, average normalized expression of genes included in these two groups was calculated after assigning them opposite values: positive (Group 1) and negative (Group 2). Its overlay on PCA maps (Figure 4D) showed gene expression gradients among seeds for each treatment that are absent in the pool-split negative control. In addition, seed sub-pools (Figure 3B) showed significant differences in that signature values, for each time point, except for pool-split control (Figure 4E). Concordantly with seed variability analysis (Figure 3), the difference between seeds’ sub-pools was smallest for the 7-day time point and largest for the recovery time point. However, it is important to stress that despite decreasing variability, the differences between seed sub-pools after 7 days of treatment can still be detected.

Analysis of the single-seed transcriptomes during the SD induction time course revealed co-expressed gene groups whose expression reflects transcriptional competence to germinate. Expression of those gene groups separates experimental time points and, at least to some degree, also positions single seeds in each of them.

### Single-seed RNA-seq analysis of the *dog1* mutant

To explore the transcriptomic gradient of germination competence, we focused on one of the key seed-specific dormancy/germination regulators—the *DOG1* gene. Our initial physiological test showed that it is involved in SD induction (Figure 1B) as it was described earlier (Footitt et al., 2011, 2020; Buijs et al., 2020). Despite *DOG1* expression being reported to be affected upon SD induction (Footitt et al., 2011, 2015, 2020), in our single-seed RNA-seq experiment it was too noisy to be analyzed directly, perhaps because the *DOG1* gene is on the edge of the detection threshold (Supplemental Figure S7A). Limited detection of lowly expressed genes is immanent property of both single seed and single-cell RNA-seq approaches. To overcome this obstacle, we first used an indirect strategy, whereby mRNAs from pools of *dog1-4* mutant and Col-0 dry after-ripened seeds were sequenced. We identified 372 downregulated and 290 upregulated genes (false discovery rate – FDR < 0.05; DESeq2) (Supplemental Figure S7B), which show GO term enrichment for response to abiotic stimulus, including heat stress, water deprivation, and hypoxia (Supplemental Figure S7C). We created a gene expression signature (Vision package) based on *dog1-4*-affected genes that revealed the degree of similarity of seeds during SD induction to *dog1-4* mutant. Globally co-regulated Group 1 (germination-associated) genes and *dog1-4*-affected genes showed a highly similar pattern (Figures 4, A and 5, A) and high correlation among single seeds (Pearson correlation 0.92) despite the limited overlap between these sets of genes (Supplemental Figure S7D). Interestingly, half of Group 2 (dry seed-associated) genes were found among *dog1-4* DEGs (Supplemental Figure S7D). In accordance, Group 2 (dry seed-associated) genes also showed a strong, albeit negative correlation with the *dog1-4* signature (Pearson correlation −0.89). In general, expression of DOG1-regulated genes is reverse to the composite pattern of germination competence: high in 1 h time point, decreasing in 1 day, increasing gradually in 3, 5, and 7 days and diminishing in 7 days + 24 h, with heterogeneity among the two seed sub-pools. As DOG1 is a well-known positive regulator of dormancy, this signature supports our hypothesis that the identified gene co-expression groups reflect competence for germination.

In a more direct approach, we performed single-seed RNA-seq for Col-0 and *dog1-4* mutant for two time points: 3 days and 7 days + 24 h (96 seeds for each genotype and condition—384 seeds in total). Counts were filtered as above, including removing genes whose expression was correlated with the intergenic reads (9,110 genes and 382 seeds remaining) (Supplemental Figure S8, A–E). Seurat/sctransform (Hafemeister and Satija, 2019; Hao et al., 2021) analysis of data showed grouping of seeds according to time points (Figure 5B) which seems to be not associated with the number of reads, intergenic reads, identified genes, or seed batches (Supplemental Figure S8, F–I). Differential gene expression for 3 days and 7 days + 24 h time points provided 27 and 233 DEGs, respectively (Supplemental Figure S9A; Supplemental Data Set 2), showing that stronger differences between genotypes appear during seed recovery from SD inducing conditions.

Our analysis of genes co-regulated in Col-0 plants led us to propose that during SD induction, seeds are positioned on a single transcriptomic path (Figure 4C). Therefore, we prepared a similar analysis for our single-seed RNA-seq in Col-0 and *dog1-4* seeds at 3 days and 7 days + 24-h time points. Again, the expression of the two most numerous globally co-regulated gene groups was negatively related (Supplemental Figure S9, B and C), with the most numerous group showing a substantial overlap with Group 1 (germination-associated) from the first experiment (Supplemental Figure S9D). Albeit globally co-regulated Group 2 from both experiments show much weaker overlap (Supplemental Figure S9E), they create a similar pattern on the PCA map and show a high correlation (0.97; Supplemental Figure S9F). The direct comparison of signature values for the two main gene groups again indicates a transcriptional gradient of germination competence (Figure 5C). In agreement with the published role of DOG1 protein as a specific positive regulator of seed dormancy (Bentsink et al., 2006) and the strong requirement for DOG1 to enter into dormancy in our protocol, our data show that *dog1-4* mutant seeds align to the same gradient and are more advanced toward germination than Col-0 seeds.

Analysis of *dog1* mutant validates the single-seed RNA-seq as a method capable of detecting seed heterogeneity. It confirms that *DOG1* is a major player in the control of seed biology during SD establishment and validates the importance of transcriptomic germination competence.

## Discussion

### Method

Analysis of single-seed transcriptomes has several advantages when compared to seed pools experiments. In traditional RNA-seq experimental set-ups, natural gene expression variability between individual seeds is lost. Given the known heterogeneity in seeds’ response to a range of environmental factors (Mitchell et al., 2016), including bet-hedging phenomena, we envisage that the single-seed RNA-seq method will be useful whenever the physiological behavior is not fully penetrant. Importantly, it allows the identification of seed populations behaving differently from the majority of studied seeds before any visible phenotypes can discriminate them. In our SD induction experiment, we identified sub-populations of seeds characterized by distinct gene expression profiles that are most evident for the 7 days + 24-h time point (Figure 3). The use of pool-split control allowed us to estimate the level of technical variability inherent to any measurements (Figure 3). Moreover, the usage of a large number of data points enhanced the identification of co-expressed gene groups driving seeds heterogeneity (Figure 4). In addition to studies of variability, our single-seed RNA-seq method can be used to analyze transcriptomes in seeds of mutants with early seedling lethality.

We compiled our single-seed RNA-seq protocol from BRB-seq and other similar methods (Hennig et al., 2018; Alpern et al., 2019), making it cost- and time-effective and enabling the study of hundreds of single-seed Arabidopsis transcriptomes. To our knowledge, only experiments with column-based isolation of RNA from dozens of single Arabidopsis seeds and subsequent RT-qPCR analysis have been described in the literature (Tao et al., 2017). Applying a few hundred isolations to obtain high-quality RNA would make single-seed analysis too expensive and time-consuming to be done in most laboratory settings. That is why we decided to perform reverse transcription reactions directly from seed extracts which enable fast parallel processing of many seeds. Seed extracts are pretreated with nonionic detergents and proteinase K to make mRNAs more accessible to the enzyme. However, suboptimal purity of RNA and buffering conditions make individual RT reactions less efficient, in consequence limiting the detection range of the method (excluding lowly expressed genes) and prohibiting from direct analysis many important regulatory factors. Further improvements of the method could include optimization of seed extract preparation and reverse transcription conditions. It may also include the addition of spike-in transcripts to better estimate reverse transcription efficiencies and the amount of mRNA available in each seed (Brennecke et al., 2013). Given the small size of Arabidopsis seeds, we envisage that our method can be applied to any seed, but due to species differences in seed size, morphological, and storage material characteristics, it may be necessary to optimize the seed extract preparation for successful single-seed RNA-seq.

### Transcriptional competence to germination

Using our single-seed RNA-seq, we analyzed transcriptomic diversity during SD induction. On a global scale, gene expression changes during time course can be attributed to seed imbibition, response to persisting stress, and stress recovery. In all these cases, the expression of translation-related genes was among the most affected (Figure 2). Interestingly, translation-related genes were recently found to differentiate dormant and nondormant seeds (Dekkers et al., 2016a, 2016b; Wilhelmsson et al., 2019), to be upregulated during seed imbibition (Bai et al., 2017) and affected during dormancy cycling (Buijs et al., 2020). As a result, it has been suggested that the upregulation of translation-related genes is an early hallmark of germination (Buijs et al., 2020). The importance of this set of genes for SD induction and recovery is corroborated by their strong enrichment among the globally co-regulated gene Group 1 (germination-associated). In contrast, Group 2 is mostly composed of genes characteristic of dry seeds.

We took advantage of the two identified antagonistically behaving gene groups to infer the position of seeds on the transcriptomic gradient of germination competence (Figure 4). It is important to stress that the absolute value of transcriptomic germination competence does not determine if the seed will become dormant or not. Seeds from the 1-h time point all have low transcriptomic germination competence but germinate when transferred to permissive conditions because they lost primary dormancy. This shows that germination competence describes the current transcriptomic state of the seed. As a result, when different time points are compared, it allows for estimation of the proximity of the seed population to germination (Figure 6). Importantly, we observe relative differences of these values inside each time point tested, which suggests it allows for the identification of seeds leaning to germination or dormancy in any seed pool. Therefore, we hypothesize that transcriptomic germination competence may directly or indirectly reflect the relative position of individual seeds along with sensitivity thresholds distribution in the population as proposed in PBT models (Bradford, 2018).

This hypothesis was supported by an analysis of the mutant in the *DOG1* gene, which has been described as a key regulator of seed dormancy in Arabidopsis and a gene under strong evolutionary selection (Bentsink et al., 2006). We observed a similarity of *dog1-4* mutant signature with transcriptomic germination competence as well as a clear shift of the *dog1-4* mutant seeds along this gradient (Figure 5). Both observations support the interpretation of the heterogeneity among wild-type seeds as a difference in germination capability. Moreover, the fact that *dog1-4* seeds align with this gradient reinforces the view that DOG1 controls seed features that naturally vary among seeds, as suggested by a recent quantitative trait locus analysis (Abley et al., 2021).

Finally, a meta-analysis of publicly available microarray data from a set of mutants, ecotypes, and biological treatments (Bassel et al., 2011; Dekkers et al., 2016a, 2016b) has previously identified seed-specific co-expressed gene clusters, and two of these clusters also show the potential to separate single seeds along a single gradient (Supplemental Figure S9, G and H). To sum up, our results show that the use of single-seed RNA-seq reveals continuous gradients of gene expression, underpinning nonbinary variability between individual seeds.

### Seed variability

Seed variability is a natural phenomenon that forms the basis of a bet-hedging strategy allowing diversification of the response to the external environment (Mitchell et al., 2016). It underlies the survival of seeds in an unpredictable natural environment, allows dispersal, the persistence of soil seed banks (Footitt et al., 2020), seed heteromorphy (Lenser et al., 2016), and many other seed related processes. Except for a study on individual seedlings’ variability during the diurnal cycle (Cortijo et al., 2019), few studies have addressed the organismal-level transcriptional variability in plants. However, more work was performed on the level of single cells, including the pioneering work showing cell size-dependent variability of *FLOWERING LOCUS C* (a key flowering time regulator) expression in root cells (Ietswaart et al., 2017). Recent advances in single-cell RNA sequencing have allowed in-depth cataloging of cell types and suggested variability between them (Picard et al., 2021). In agreement, live imaging of transcription in plants (Alamos et al., 2021; Hani et al., 2021) has shown considerable heterogeneity even between cells of the same type. Similarly, condensate formation on the level of single-seed cells was proposed to contribute to bet-hedging phenomena (Dorone et al., 2021). However, it is unclear how the single-cell variability can be integrated among the cells to result in organism-level responses.

Our method was designed to examine gene expression variability among Arabidopsis seeds. Applying this strategy to the analysis of the SD induction time course led us to propose specific sources of seed variability at studied time points that overlay on initial seed pool heterogeneity. These include a difference in the rate of water uptake (1 h), competition between imbibition induced germination and stress conditions inhibiting it (1 day), response to stress (3, 5, and 7 days), seed recovery, and the start of the germination (7 days + 24 h). Interestingly, seed variability decreases in 3- and 5-day time points to reach a minimum in 7 days, but still exceeds the pool-split control (Figures 3 and 6—Model). The highest variability may be observed for the 7 days +24 h time point. In our experiment, we observed that up to 50% of seeds germinate despite 7 days of SD induction (Figure 1). In contrast to this binary outcome, we observe a continuous transcriptomic gradient among the 7 days + 24-h time point Col-0 seeds (Figure 5). A partially overlapping gradient is also observed for *dog1-4* mutant seeds, for which we showed almost 100% germination (Figure 1B). These observations imply that 24-h time point is too early to establish a clear binary transcriptomic outcome of treatment. It also suggests a continuous distribution of germination competence in the seed population that is shifted upon SD induction. This conclusion is in agreement with different percentages of seeds germinating when exposed to varying temperatures during the recovery phase (Supplemental Figure S1).

All these results may also be interpreted in the frame of PBT models in which the speed of germination is proportional to the distance to the sensitivity threshold. We expect that the clear cutoff between seeds would be evident only after a period in which all seeds whose sensitivity thresholds were achieved will start the germination process. According to PBT models, germination of an individual recovering seed is an on/off decision that results from the underlying continuous distribution of sensitivity thresholds. Our data suggest that sensitivity thresholds may be reflected by the continuous transcriptomic gradient of germination competence that could be at least partially preserved during strong and prolonged stress conditions. This ability to maintain heterogeneity in their competence to germinate may be a key feature of soil seed banks where despite the repeated cycles of dormancy induction and release, not all of the seeds commit to germination during vegetation seasons (Warr et al., 1994; Evans et al., 2007). The direction of a causative link between sensitivity thresholds, germination competence and observed gene expression patterns is difficult to determine due to reciprocal influence on each other. However, future experiments using our single-seed RNA-seq method together with mutant analysis can help to solve that problem.

### Establishment of germination status

It has been suggested that pools of freshly harvested (dormant) and after ripened (nondormant) seeds can be transcriptionally distinguished only during seed imbibition and not in the dry state (Dekkers et al., 2016a, 2016b). We expected that processes underlying the regulation of germination would be more evident in the imbibed than dry seeds. Our experiment was designed to observe gradual changes in seed transcriptomic profiles leading to binary germinating/nongerminating phenotypes of seeds after SD induction treatment. Persistent stress conditions reduced seeds heterogeneity during the treatment, and strong transcriptomic gradients appeared only during seed recovery, which can be interpreted in different ways. In terms of PBT models, it is a question about the exact moment when sensitivity thresholds are established and when they start to affect the seed’s physiology.

Seeds sensitivity thresholds may be established during SD induction, but our method fails to capture it. Perhaps this failure is due to the depth of sequencing or because thresholds are based on the differential accumulation of metabolites or oxidation state changes that do not immediately affect mRNA levels. So that means that thresholds would be set during dormancy induction but revealed at the transcriptional level only during the recovery stage. Alternatively, differential sensitivity is set upon stress alleviation (Figure 6—Model). This is supported by the fact that seed germination competence is strongly increased in all seeds during recovery, albeit to a varying degree. It means that even seeds leaning toward dormancy modify their transcriptomes during recovery. The establishment of sensitivity thresholds and resulting germination competence may be assisted by several different quality checkpoints needed to estimate the seed’s physiological state after stress conditions ended. The nature of these checkpoints is not yet clear, but it has been suggested that DNA damage status is one of the important aspects controlled during seed imbibition (Waterworth et al., 2016). We favor the possibility that while stress treatment affects the span of variability between individual seeds, the probability of single-seed germination is also influenced by earlier and later events (Figure 6—Model). It is even possible that the likelihood of germination may be affected at the stage of seed development and primary dormancy establishment (Auge et al., 2015). In that interpretation, seed relative position along the pattern of transcriptional germination competence is maintained throughout the SD induction time course.

## Conclusions

Here, we demonstrate a method for analysis of hundreds of single Arabidopsis seed transcriptomes which could boost studies of organismal variability and bet-hedging phenomenon. It could also find application in the analysis of early events in plant life like seed maturation and germination. We used single-seed RNA-seq for assaying gene expression changes and differences between single seeds upon SD induction. Our results show that several co-expressed gene groups create specific patterns during the SD induction time course. Translation-related transcripts are the most prominent of these gene groups, and our data suggest that they are important markers of seed germination competence that reflect the distribution of sensitivity thresholds of PBT models (Bradford, 2018). Notably, their expression also shows a decrease in seed heterogeneity during the time-course of the treatment and its increase in germination-promoting conditions. Our observation suggests that seed transcriptomic variability may persist in strong stress conditions allowing for the re-establishment of phenotypic diversity upon recovery.

Seeds form the basis of agriculture as well as a vital component of plant survival in nature. Heterogeneity among seeds is a well-known phenomenon with numerous ecological, physiological, and theoretical studies addressing its importance (Bradford and Trewavas, 1994; Mitchell et al., 2016; Bradford, 2018). Our single-seed RNA-seq method is able to reveal hidden variability in uniform seed pools and may be used to provide the molecular basis of seed biology.

## Materials and methods

Col-0, *dog1-4* (Bentsink et al., 2006), and *nced2/5/6/9* (Chauffour et al., 2019) seeds were used. To obtain seeds for experiments, Arabidopsis (*A.thaliana*) plants were grown in a greenhouse in a long-day photoperiod at 22°C. Seeds combined from two plants were used as a single biological replicate. For SD induction, dry seed pools (stored for at least 6 months) were tested for residual primary dormancy, and seeds were later sown on plates with water-soaked blue paper. Plates were sealed with parafilm and incubated in the dark at 30°C for 1, 3, 5, or 7 days (as indicated in figures and text). Seed recovery and germination tests were performed by placing plates in constant light at 22°C (or other indicated temperatures). Seed stratification was performed for 2 days at 4°C.

For single-seed RNA-seq, 16 seeds were sparsely placed on a cooled microscopic glass slide and crushed using another slide. An aliquot of 2 µL of cold water was quickly added to each seed debris on both slides. The matching two parts of each seed debris were mixed with water by pipetting and combined. An aliquot of 3 µL of seed extract was transferred to the PCR tube on ice. For pool and split control, all seed extracts were combined into a single tube, mixed, and split into 3 µL aliquots. After all seeds were processed, 2 µL of digestion buffer (10-mM Tris–HCl pH 8.0, 1% Triton X-100 (v/v), 1% Tween-20 (v/v), 0.5-mM EDTA, 1.3 mg/mL proteinase K), and 1 µL of 10-mM barcoded oligo(dT) primer (Supplemental Table S2) were added and mixed. Samples were incubated for 10 min at 65°C and 1 min at 85°C and placed on ice. Digested extracts were mixed with 4 µL of reverse transcription mix (2-mM dNTPs, 2× SuperScriptIII reaction buffer, 10 mM DTT, 50 U of SuperScriptIII, 10 U of RiboLock), incubated for 30 min at 42°C, 15 min at 70°C, and placed on ice. About 32 cDNAs obtained using different barcoded oligo(dT) primers (Supplemental Table S2) were pooled together, purified with Ampure beads (1.5× beads to sample volumes), and eluted in 17 µL of water. An aliquot of 15 µL of recovered cDNA pool was mixed by pipetting with 15 µL of second-strand synthesis mix (2× NEBNext Second Strand Synthesis (dNTP-free) Reaction Buffer (NEB, Ipswich, MA, USA), 1 U RNaseH (NEB), 1 U *Escherichiacoli* DNA ligase I (NEB), 5 U *E. coli* DNA polymerase (NEB), 30 µM dNTPs) and incubated overnight at 16°C. Reactions were purified with AMPure XP beads (Beckman Coulter, Indianapolis, IN, USA) (1.2× beads to sample volumes) and eluted in 4 µL of water. Tn5 enzyme was obtained as in Hennig et al. (2018), loaded with B duplex and diluted 5 times. An aliquot of 3 µL of recovered dsDNA sample was used for tagmentation using 3 µL diluted Tn5 enzyme and 6-µL freshly prepared 2× buffer (20-mM Tris–HCl pH 7.5, 20-mM MgCl_2_, 50% dimethylformamide (DMF). Reactions were mixed by pipetting and incubated for 7 min at 55°C followed by 5 min at 80°C. Water was added up to 30 µL, samples were purified with Ampure beads (1.2× beads to sample volumes) and eluted with 11 µL of water. Illumina indexing PCR was performed using 10 µL tagmented DNA in 50 µL reactions with Q5 2× PCR Master Mix (NEB) and 0.5-µM final concentration of primers. To avoid PCR over-cycling, we estimated the number of cycles as in Buenrostro et al. (2015). Libraries were sequenced on Illumina NextSeq 500 system using the paired-end mode to obtain 21 nt R1 (containing barcode and UMI) and 55 nt R2 (containing mRNA sequence).

After quality control using fastqc, reads R1 and R2 were processed separately. In our oligo(dT) primers, two parts of UMI are split by a barcode sequence (BC), that is why we transformed read R1 fastq file using awk command: *awk ‘NR%2==1 {print $0} NR%2==0 {print substr($1,16,5) substr($1,1,15)}’*. Read R2 was trimmed to remove potential contamination with poly(A) tail using BRBseqTools (version 1.6) Trim (Alpern et al., 2019) and parameters *-polyA 10 -minLength 30*. R2 reads were mapped using STAR (version 2.7.8a; Dobin et al., 2013) to TAIR 10 genome version and Araport11 genome annotation with parameters *–sjdbOverhang 54 –outSAMtype BAM SortedByCoordinate –outFilterMultimapNmax 1*. Finally, the count matrix for each seed and each gene was obtained using BRBseqTools (version 1.6) CreateDGEMatrix (Alpern et al., 2019) with parameters *-p UB -UMI 14 -s yes*, using Araport11 genome annotation and a list of barcodes. Count matrixes from different libraries were combined and used for further analysis using the Seurat R package (Hafemeister and Satija, 2019; Hao et al., 2021).

For transcriptomic analysis of dry seed pools of Col-0 and *dog1-4*, RNA from three biological replicas was isolated using a standard protocol (Meng and Feldman, 2010). RNA was treated with Turbo DNase (ThermoFisher, Waltham, MA, USA), and 500 ng of it was used in reverse transcription using 50-mM different barcoded oligo(dT) primers and SuperScript III. About 0.25 of each reaction was pooled, and the library was prepared and sequenced exactly as above. Read R1 was transformed as above and read R2 was processed as follows. Information about UMI was put to read R2 name using umi_tools (version 1.1.0) extract (Smith et al., 2016) with parameter –bc-pattern=NNNNNNNNNNNNNNCCCCCC. Then fastq file for read R2 was divided into separate fastq files for each replica using BRBseqTools (version 1.6) Demultiplex with parameters -p UB -UMI 14. Each file was then trimmed and mapped as for single-seed libraries. Finally, mapped reads were deduplicated using umi_tools (version 1.1.0) dedup (Smith et al., 2016) with parameter—spliced-is-unique. Counts quantified for genes of Araport11 genome annotation using htseq-count (version 0.11.3; Anders et al., 2015) were used for differential gene expression analysis using the DESeq2 R package (Love et al., 2014). Scripts for generation of figures for the manuscript are available at Github https://github.com/mk1859/single_seed.

### Accession numbers

The datasets generated and analyzed during the current study are available in the GEO repository under accession number GSE185033.

## Supplemental data 

The following materials are available in the online version of this article.

**Supplemental Figure S1.** Sensitivity of seeds after different time points of dark and high-temperature treatment (0, 1 day, 3 days, and 7 days) to varying temperatures during recovery.

**Supplemental Figure S2.** Quality controls for single-seed RNA-seq of SD induction time course.

**Supplemental Figure S3.** Properties of PCA map of SD induction time-course experiment.

**Supplemental Figure S4.** Seeds show the highest transcriptome variability at the recovery time point and lowest at the final time point of stress treatment.

**Supplemental Figure S5.** Single-seed RNA-seq expression profiles for selected genes important for seed biology and hormonal germination regulation.

**Supplemental Figure S6.** Maps of remaining globally co-expressed gene groups.

**Supplemental Figure S7.** Differential gene expression between Col-0 and *dog1-4* after ripened dry seeds.

**Supplemental Figure S8.** Quality controls of single-seed RNA-seq for SD induction in Col-0 and *dog1-4* mutant seeds.

**Supplemental Figure S9.** Gene expression signatures allow aligning *dog1-4* mutant seeds according to germination competence.

**Supplemental Table S1.** The variance of gene expression signatures among seeds from different time points.

**Supplemental Table S2.** Primers used for single-seed RNA-seq.

**Supplemental Data Set 1.** Results of the single-seed RNA-seq time-course experiment.

**Supplemental Data Set 2.** Results of the RNA-seq for *dog1-4* and Col-0 seed pools and *dog1-4* and Col-0 single-seed RNA-seq experiment.

## Supplementary Material

kiac265_Supplementary_Data

## Notes

M.K. performed single-seed RNA-seq experiments and analyzed all the data. R.Y. and M.W. established the secondary dormancy induction protocol and performed physiological tests. S.P.S. prepared a seed pool RNA-seq experiment. D.A. and M.K. designed the single-seed RNA-seq library preparation method. S.S. supervised all experiments. S.S. and M.K. wrote the manuscript. All authors read and approved the final manuscript.

The author responsible for distribution of materials integral to the findings presented in this article in accordance with the policy described in the Instructions for Authors (https://academic.oup.com/plphys/pages/general-instructions) is Szymon Swiezewski (sswiez@ibb.waw.pl).
